# L-lactate induces specific genome wide alterations of gene expression in cultured bovine granulosa cells

**DOI:** 10.1186/s12864-019-5657-6

**Published:** 2019-04-05

**Authors:** Anja Baufeld, Dirk Koczan, Jens Vanselow

**Affiliations:** 1 Institute of Reproductive Biology, Leibniz Institute for Farm Animal Biology (FBN), Wilhelm-Stahl-Allee 2, 18196 Dummerstorf, Germany; 2 Institute for Immunology, University of Rostock, 18055 Rostock, Germany

**Keywords:** Tissue culture, Gene expression, Signaling pathways, mRNA microarray, Angiogenesis

## Abstract

**Background:**

Previously, we could show that L-lactate affects cultured bovine granulosa cells (GC) in a specific manner driving the cells into an early pre-ovulatory phenotype. Here we studied genome wide effects in L-lactate-treated GC to further elucidate the underlying mechanisms that are responsible for the L-lactate induced transformation. Cultured estrogen producing GC treated either with L-lactate or vehicle control were subjected to mRNA microarray analysis.

**Results:**

The analysis revealed 487 differentially expressed clusters, representing 461 annotated genes. Of these, 333 (= 318 genes) were identified as up- and 154 (= 143 genes) as down-regulated. As the top up-regulated genes we detected *TXNIP*, *H19* and *AHSG* as well as our previously established marker transcripts *RGS2* and *PTX3.* The top down-regulated genes included *VNN1*, *SLC27A2* and *GFRA1*, but also *MYC* and the GC marker transcript *CYP19A1*. Pathway analysis with differentially expressed genes indicated “cAMP-mediated signaling” and “Axon guidance signaling” among the most affected pathways. Furthermore, estradiol, progesterone and Vegf were identified as potential upstream regulators. An effector network analysis by IPA provided first hints that processes of “angiogenesis” and “vascularization”, but also “cell movement” appeared to be activated, whereas “organismal death” was predicted to be inhibited.

**Conclusions:**

Our data clearly show that L-lactate alters gene expression in cultured bovine GC in a broad, but obviously specific manner. Pathway analysis revealed that the mode of L-lactate action in GC initiates angiogenic processes, but also migratory events like cell movement and axonal guidance signaling, thus supporting the transformation of GC into an early luteal phenotype.

**Electronic supplementary material:**

The online version of this article (10.1186/s12864-019-5657-6) contains supplementary material, which is available to authorized users.

## Background

Folliculogenesis is a finely tuned process of cellular differentiation. The most crucial step of differentiation is the folliculo-luteal transition that is initiated by the pre-ovulatory LH surge. Besides the release of a fertilizable oocyte in particular in the bovine this transition involves a deep transformation of somatic cells of the follicular wall into luteal cells. This is essential for regulating the ovarian cycle and supporting an ongoing pregnancy. In this transition phase the follicle is completely remodeled from the vesicle-like estradiol (E2)-producing dominant follicle into the compact progesterone (P4)-producing corpus luteum (CL). In the bovine, cells of the granulosa and theca layers migrate and largely intermingle with each other during CL formation [1]. This remodeling of the follicle is preceded and accompanied by a profound and meticulous regulation of gene expression in particular in the granulosa cell layer. Especially genes involved in steroidogenesis have been shown to be strongly regulated by the LH surge [2–5]. *CYP19A1*, encoding the key enzyme of estradiol synthesis (aromatase) is massively down-regulated together with the gonadotropin receptors *FSHR* and *LHCGR*. On the other hand several genes are highly up-regulated due to LH, namely *RGS2* (regulator of G protein signaling 2), *VNN2* (vanin 2) and *PTX3* (pentraxin 3). *VNN2* and *PTX3* are involved in processes of inflammation. Moreover, *PTX3* has been demonstrated to be essential for female fertility by organizing stable extracellular matrix architecture for an intact cumulus oophorus complex [6–8]. *RGS2* interacts with the Gα subunit of G proteins by blocking Gα-mediated signaling [9] and was shown to modulate LH receptor signaling thus having an important role during the folliculo-luteal transition [10, 11]. From this knowledge, typical markers for LH-dependent differentiation in the bovine could be established [3], whereby in particular the role of various growth-factors as those of the TGFbeta superfamiliy or EGF and their role during follicle differentiation were analyzed in detail [12–15]. In our previous study we could demonstrate that L-lactate, a molecule that is usually known to be connected to the energy metabolism, can act as a signaling molecule that specifically influences gene expression and thus remarkably influences GC differentiation in vitro [16]. Other studies could show that L-lactate is present at much higher levels within the follicular fluid than in serum [17, 18]. Additionally, it was shown that the L-lactate levels in rats increase at the expected time of the LH-surge implicating a regulatory role that forces GC differentiation [19]. During the present study we analyzed the effects of L-lactate in a genome wide mRNA microarray approach with subsequent bioinformatic evaluation of the datasets to elucidate the underlying pathways and biological processes.

## Methods

### Tissue collection and cell culture

Ovaries were collected at a local abattoir irrespective of age, nutritional status or stage of ovarian cycle and transported in 1x PBS (with 100 IU penicillin, 0.1 mg/ml streptomycin and 0.5 μg/μl amphotericin). For each cell preparation granulosa cell pools were obtained by aspiration of small to medium sized follicles (< 6 mm) with a syringe and 18 G needle from 30 to 40 ovaries. By follicle aspiration almost exclusively cells from the granulosa without contaminating thecal cells were collected [4]. Living cells were counted using the trypan blue exclusion method and cryo-preserved in freezing media (fetal calf serum containing 10% DMSO; Roth, Karlsruhe, Germany). Additionally, a small portion of the freshly aspirated sample-pool was preserved as “pre-cultured sample” for qPCR analysis in liquid nitrogen. For seeding, the GC were thawed and immediately transferred into α-MEM and centrifuged at 500 x g for 3 min to ensure fast removing of the freezing media. Afterwards the cell pellet was diluted in α-MEM containing L-Glutamine (2 mM), sodium bicarbonate (10 mM), BSA (0.1% *w*/*v*), HEPES (20 mM), sodium selenite (4 ng/ml), transferrin (5 μg/ml), insulin (10 ng/ml), non-essential amino acids (1 mM), penicillin (100 IU/ml) and streptomycin (0.1 mg/ml), FSH (20 ng/ml; Sigma Aldrich, Steinheim, Germany), R^3^ IGF-1 (50 ng/ml; Sigma Aldrich), and androstenedione (2 μM; Sigma Aldrich). Improved attachment of plated GC was achieved by coating the wells with Collagen R (0.02%; Serva, Heidelberg, Germany). Cells were cultured at a density of 1.0 × 10^5^ living cells per well. Cells were additionally treated with sodium L-lactate (30 mM; Sigma Aldrich), sodium chloride as vehicle control (30 mM; Sigma Aldrich) or left untreated. If not stated otherwise all reagents were purchased from Merck Millipore (Berlin, Germany). GC were cultured for 8 days at 37 °C and 5% CO_2_ and two-third of the media with or without L-lactate or vehicle was exchanged every other day.

### RNA preparation, cDNA synthesis and quantitative real-time PCR

Total RNA was isolated with the RNeasy Plus Mini Kit (Qiagen, Hilden, Germany) according to the manufacturer’s instructions. Measuring the RNA concentration was done with a NanoDrop 1000 Spectrophotometer (Thermo Scientific, Bonn, Germany). Afterwards, cDNA synthesis was performed using the SensiFAST cDNA Synthesis Kit (Bioline, Luckenwalde, Germany) from 150 ng RNA. Validation of the microarray data was accomplished with quantitative real-Time PCR (qPCR). Therefore, SensiFAST SYBR No-ROX (Bioline) was used with gene-specific primers as listed in Additional file 1: Table S1. The amplification was done in duplicate from 0.2 and 0.4 μl cDNA in a total volume of 12 μl in a LightCycler 96 instrument (Roche, Mannheim, Germany). The ensuing cycle conditions were used: pre-incubation at 95 °C for 5 min, 40 amplification cycles of denaturation at 95 °C for 20 s, annealing at 60 °C for 15 s, extension at 72 °C for 15 s and single-point fluorescence acquisition for 10 s. At the end of each run, the melting point was analysed to check for amplification of the right products. Additionally, PCR products were checked by agarose gel electrophoresis (3%, stained with Roti-GelStain, Roth, Karlsruhe, Germany). As external standards for quantification cloned and sequenced products were used. Therefore, five different dilutions of the respective standards (5 × 10^− 12^ – 5 × 10^− 16^ g DNA/reaction) were freshly prepared and co-amplified. To check for appropriate reference genes in this experimental setup the following commonly used genes were examined: *B2M* (Beta-2-microglobulin), *GAPDH* (Glyceraldehyde-3-phosphate dehydrogenase), *RPLP0* (Ribosomal protein lateral stalk subunit P0) and *TBP* (TATA box binding protein). The two most stable reference genes were obtained by using the geNORM algorithm implemented in the NormqPCR package for R [20], revealing *TBP* and *B2M* as the most stable genes. For normalization the geometric mean of both was used.

### Microarray profiling, bioinformatic evaluation and statistics

The RNA of samples from the three different culture conditions (untreated, L-lactate and vehicle control, 5 samples per group, *n* = 15) were subjected to mRNA microarray analysis. The quality of RNA was checked in a Bioanalyzer Instrument (Agilent Technologies, St. Clara, CA, USA) revealing a RIN factor ranging from 9.5 to 9.9 thus showing negligible degradation of individual samples. The Bovine Gene 1.0 ST Array (Affymetrix, St. Clara, CA, USA) was used for the analysis. Amplification, labelling and hybridization was performed with the “GeneChip Expression 3’ Amplification One-Cycle Target Labeling and Control Reagents” (Affymetrix) according to the manufacturer’s protocol. Hybridization was done overnight in the GeneChipR Hybridization Oven (Affymetrix) and visualized with the Affymetrix GeneChip Scanner 3000. Raw data were processed with the Expression Console (V1.4.1.46; Affymetrix) for normalization, background reduction and a gene-level summary using the RMA method (Robust Multichip Average). Additionally, a principal component analysis (PCA) was performed and plotted in R [21]. Results of the array have been submitted to the GEO database (GSE121408). Subsequent analysis was done with the Transcriptome Analysis Console 3.0 (TAC3.0, Affymetrix) to check for differentially expressed genes under different conditions. Analysis of Variance (ANOVA) was used to calculate the *p*-value and was additionally corrected for FDR (False Discovery Rate, Benjamini-Hochberg method) integrated in TAC3.0. Levels of significance for differentially expressed genes were set at fold change (FC) > |1.5|, ANOVA *p* < 0.05 and FDR < 0.05.

Statistic evaluation of qPCR values was performed with the SigmaPlot 11.0 Statistical Analysis System (Jandel Scientific, San Rafael, CA, USA). Threshold for statistical significance was set at *p* < 0.05.

Bioinformatic analysis was done with Ingenuity Pathway Analysis (IPA, Qiagen) using a modified list of the treatments L-lactate vs. vehicle control containing 2429 transcript clusters (FC > |1.2|, *p* < 0.05, FDR < 0.05). Out of the modified list 2193 genes could be mapped in IPA, settings for the pathway analysis were restricted to genes with FC < |1.5|, p < 0.05, FDR < 0.05. All genes having a FC between |1.2| and |1.5| were characterized as abundant in the dataset but did not influence the pathway analysis.

## Results

**Fig. 1:**
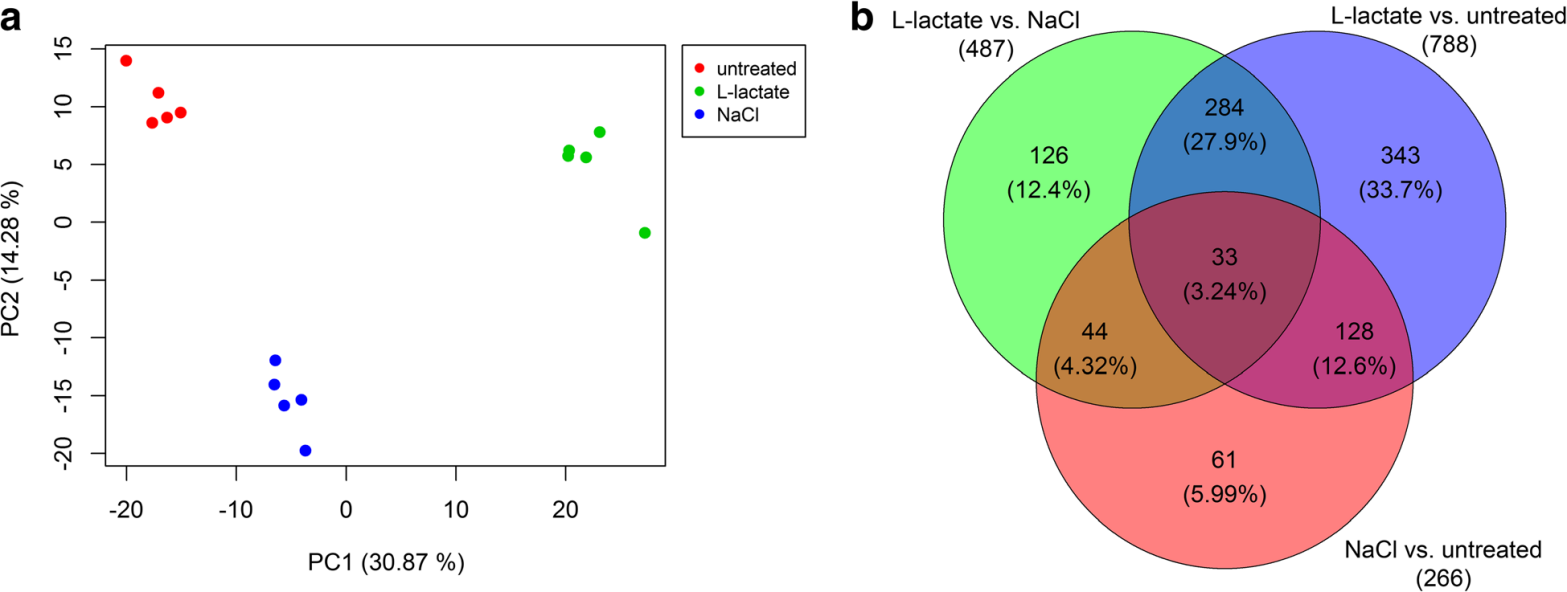
Principal component analysis (PCA) and Venn diagram of the microarray datasets. **a** PCA revealed a distinct separation of GC samples under all treatment conditions, however with the highest variation between the L-lactate treated and both other groups (PC1). **b** A Venn diagram illustrating the numbers of overlapping and differentially expressed genes of all treatment groups

Raw data from the microarray analysis were initially analyzed by principal component analysis (PCA) to reduce the multidimensionality of the dataset. Individual samples of the dataset were plotted and revealed the greatest variability between the different groups of culture conditions on the x-axis with a variation of 30.9% (Fig. 1a and Additional file 1: Table S2). Individual samples of the same culture condition clustered tightly together. Although we detected a difference between the untreated GC and the cells treated with the NaCl vehicle control, the L-lactate treatment was most distant compared to both.

On the Bovine Gene 1.0 Array Chip 26,288 transcript clusters are represented. Comparing the different groups “untreated”, “L-lactate” and “NaCl” diverse numbers of differentially expressed clusters can be observed (Fig. 1b). The higher number (788) of differentially regulated clusters (representing 735 annotated genes) of the L-lactate treatment compared to the untreated cells is in line with the PCA, demonstrating strongest effects between L-lactate treatment compared to both controls (“untreated” and “NaCl”). Least changes were observed between “untreated” and “NaCl” vehicle treated cells. Comparing L-lactate vs. NaCl vehicle control treatment revealed 487 affected clusters (representing by 461 annotated genes).

### Validation with qPCR

**Fig. 2:**
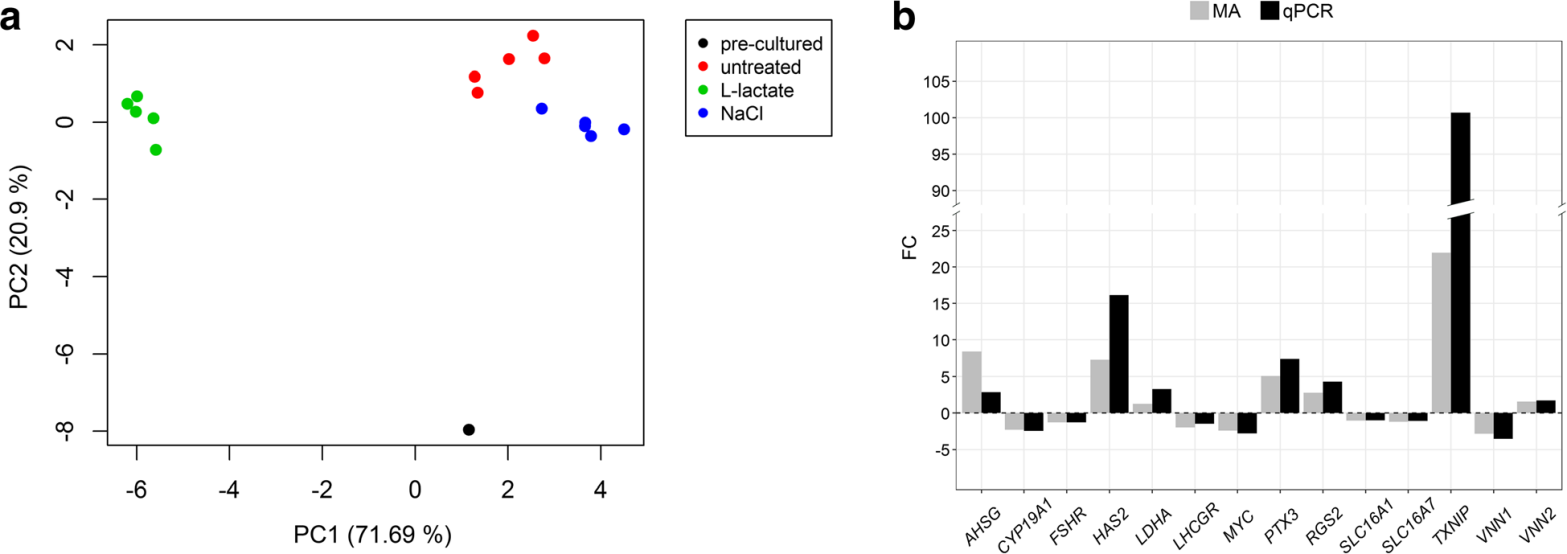
Validation of the microarray data from selected marker genes of the folliculo-luteal transition with quantitative real-time PCR. **a** PCA of the qPCR data showed highest variation between L-lactate treated and both control groups. Untreated and vehicle control treated cells cluster tightly together. The pre-cultured sample (black dot) displayed the second highest variation (PC2) to the cultured cells. **b** L-lactate vs. NaCl treated cells revealed similar fold changes in the microarray and qPCR dataset

To validate the mRNA microarray expression datasets qPCR analysis of selected transcripts was performed. Except for *SLC16A1* and *SLC16A7*, which both are not significantly regulated by L-lactate, nearly all selected genes analysed, showed high correlations between the qPCR and microarray datasets (Table 1). Principal Component Analysis of the qPCR dataset revealed the highest variation of 71.7% between the L-lactate treatment and both other culture conditions “NaCl” and “untreated” controls (Fig. 2a and Additional file 1: Table S3), with these clustering closely together. Freshly isolated, non-cultured cells were clearly separate (Fig. 2a, black dot) from cultured samples, but clearly showed more closeness to the control than the lactate treated samples considering PC1. Differential transcript concentrations in L-lactate vs. NaCl vehicle treated controls could be validated by qPCR for all selected genes (Fig. 2b). Even the fold change for most analysed genes was similar, except for *AHSG* (8.45 vs. 2.86), *HAS2* (7.31 vs. 16.12) and *TXNIP* (21.97 vs. 100.69).

**Table 1:** Comparison of qPCR and microarray data by Pearson product moment correlation analysis

gene	correlation coefficient	*p*-Value
*AHSG*	0.953	< 0.001
*CYP19A1*	0.907	< 0.001
*FSHR*	0.546	0.035
*HAS2*	0.943	< 0.001
*LDHA*	0.608	0.016
*LHCGR*	0.702	0.004
*MYC*	0.936	< 0.001
*PTX3*	0.94	< 0.001
*RGS2*	0.922	< 0.001
*SLC16A1*	−0.0699	0.805
*SLC16A7*	−0.0206	0.942
*TXNIP*	0.997	< 0.001
*VNN1*	0.926	< 0.001
*VNN2*	0.853	< 0.001

### Differentially expressed genes

Comparing the effects of lactate treatment with the NaCl vehicle control 487 transcript clusters (= 461 annotated genes) could be identified as differentially expressed. From these, 333 transcript clusters were assigned as up- and 154 clusters as down-regulated by lactate. The top 15 up- and down-regulated genes were listed in Tables 2 and 3. The highest up-regulation was found in case of *TXNIP* with a fold change (FC) of 21.97 followed by the non-protein coding gene *H19* (FC 12.36). *PTX3*, one of our formerly established markers for pre-ovulatory differentiation, was also found under the top 15 of up-regulated genes. The most down-regulated gene was *VNN1* (FC -2.82) in contrast to *VNN2* which was up-regulated as expected according to our previous data (FC 1.58, Additional file 1: Table S4). *CYP19A1*, another important GC marker was also among the top 15 down-regulated genes (FC − 2.29). Interestingly, the dataset revealed remarkably higher scores of up-regulation (FC > 21) than down-regulation (FC > − 2.8).

**Table 2:** Top 15 up-regulated genes (L-lactate- vs. NaCl vehicle control)

Gene Symbol	Description	FC	ANOVA	FDR
*TXNIP*	thioredoxin interacting protein	21.97	1.14E-10	3.10E-07
*H19*	H19, imprinted maternally expressed transcript (non-protein coding)	12.36	8.95E-07	4.10E-05
*AHSG*	alpha-2-HS-glycoprotein	8.45	1.48E-08	4.00E-06
*ARRDC4*	arrestin domain containing 4	7.42	6.26E-10	6.94E-07
*HAS2*	hyaluronan synthase 2	7.31	4.80E-07	2.70E-05
*PTX3*	pentraxin 3, long	5.11	1.24E-07	1.20E-05
*EREG*	Epiregulin	5.05	3.57E-09	2.00E-06
*NDUFA4L2*	NADH dehydrogenase (ubiquinone) 1 alpha subcomplex, 4-like 2	4.65	3.63E-07	2.30E-05
*LTC4S*	leukotriene C4 synthase	4.54	3.33E-10	4.40E-07
*LOC104976005*	uncharacterized LOC104976005	4.47	2.47E-08	5.00E-06
*RANBP3L*	RAN binding protein 3-like	4.03	2.12E-08	4.00E-06
*NPY*	neuropeptide Y	4.03	1.90E-05	3.61E-04
*CHRNA2; LOC101908817*	cholinergic receptor, nicotinic, alpha 2 (neuronal); neuronal acetylcholine receptor subunit alpha-2-like	3.90	2.91E-10	4.18E-07
*GFRA2*	GDNF family receptor alpha 2	3.43	1.53E-11	1.45E-07
*NDP*	Norrie disease (pseudoglioma)	3.40	3.65E-08	6.00E-06

**Table 3:** Top 15 down-regulated genes (L-lactate vs. NaCl vehicle control)

Gene Symbol	Description	FC	ANOVA	FDR
*VNN1*	vanin 1	−2.82	1.31E-07	1.20E-05
*SLC27A2*	solute carrier family 27 (fatty acid transporter), member 2	− 2.68	2.00E-06	6.20E-05
*GFRA1*	GDNF family receptor alpha 1	−2.65	1.04E-08	3.00E-06
*PRPS2*	phosphoribosyl pyrophosphate synthetase 2	−2.64	5.52E-09	2.00E-06
*PPM1K*	protein phosphatase, Mg2+/Mn2+ dependent, 1 K	−2.55	1.08E-09	8.78E-07
*SLC1A5*	solute carrier family 1 (neutral amino acid transporter), member 5	−2.45	4.74E-07	2.70E-05
*RANGRF*	RAN guanine nucleotide release factor	−2.41	2.00E-06	7.30E-05
*MYC*	v-myc avian myelocytomatosis viral oncogene homolog	−2.40	8.02E-08	9.00E-06
*ATF7IP2*	activating transcription factor 7 interacting protein 2	−2.40	2.00E-06	6.90E-05
*LOC508666*	C-C motif chemokine 23	−2.36	2.19E-07	1.70E-05
*IFIT1*	interferon-induced protein with tetratricopeptide repeats 1	−2.34	2.90E-07	2.00E-05
*GPT*	glutamic-pyruvate transaminase (alanine aminotransferase)	−2.31	2.62E-07	1.90E-05
*CYP19A1*	cytochrome P450, family 19, subfamily A, polypeptide 1	−2.29	9.86E-08	1.00E-05
*SLC7A11*	solute carrier family 7 (anionic amino acid transporter light chain, xc- system), member 11	−2.27	3.46E-07	2.20E-05
*B3GALT2*	UDP-Gal:betaGlcNAc beta 1,3-galactosyltransferase, polypeptide 2	−2.20	3.15E-07	2.10E-05

### Pathway analysis by IPA

**Fig. 3:**
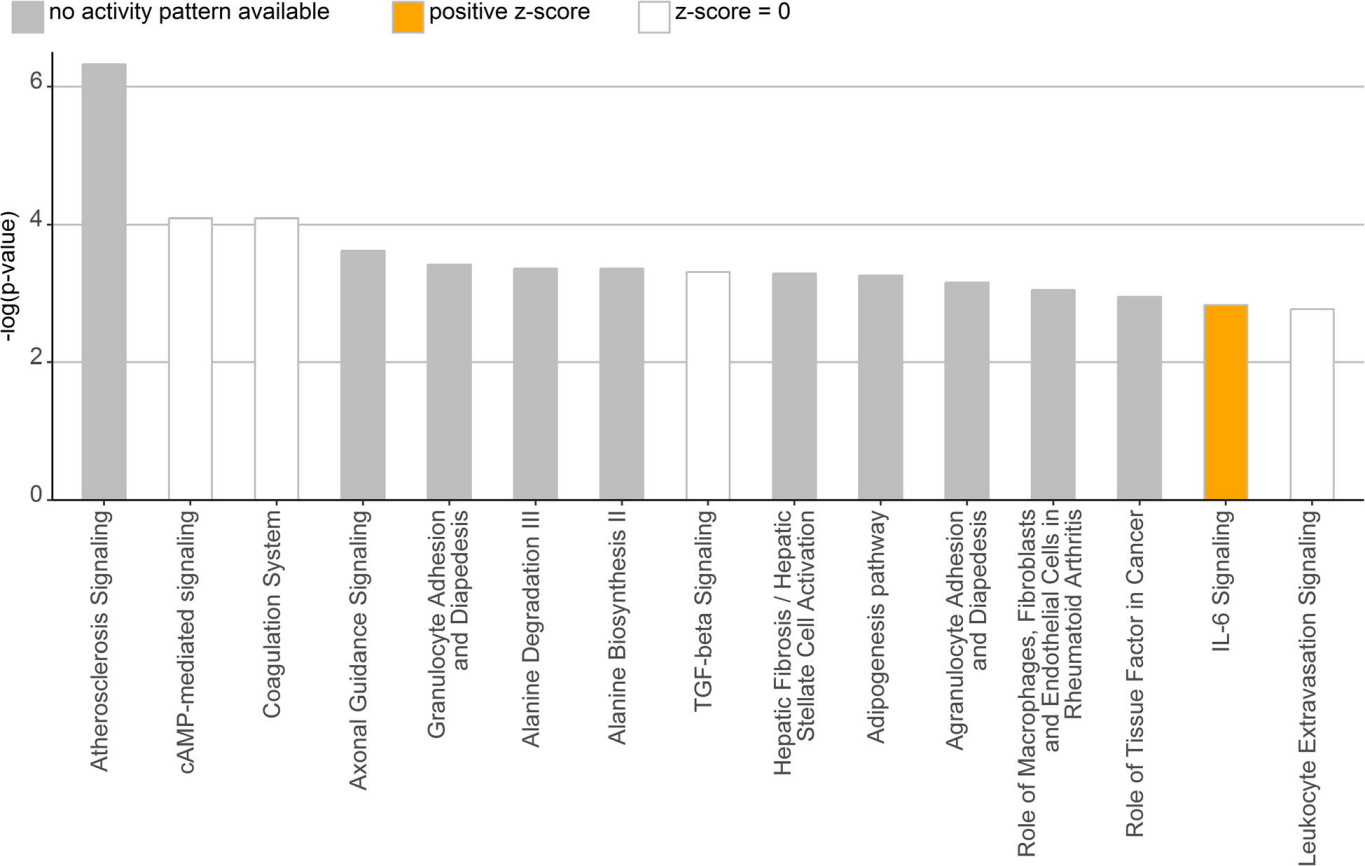
Top 15 affected pathways as indicated by IPA. Within the top affected pathways “cAMP-mediated signalling” or “Axonal Guidance Signalling” could be detected. The z-score indicates a prediction of activation or inhibition of a specific pathway based on experimentally determined gene expression data and the IPA ‘knowledge base’. A positive z-score (activation) was only predicted for IL-6 Signaling. But for most of the pathways no prediction regarding activation or inactivation could be made

**Fig. 4:**
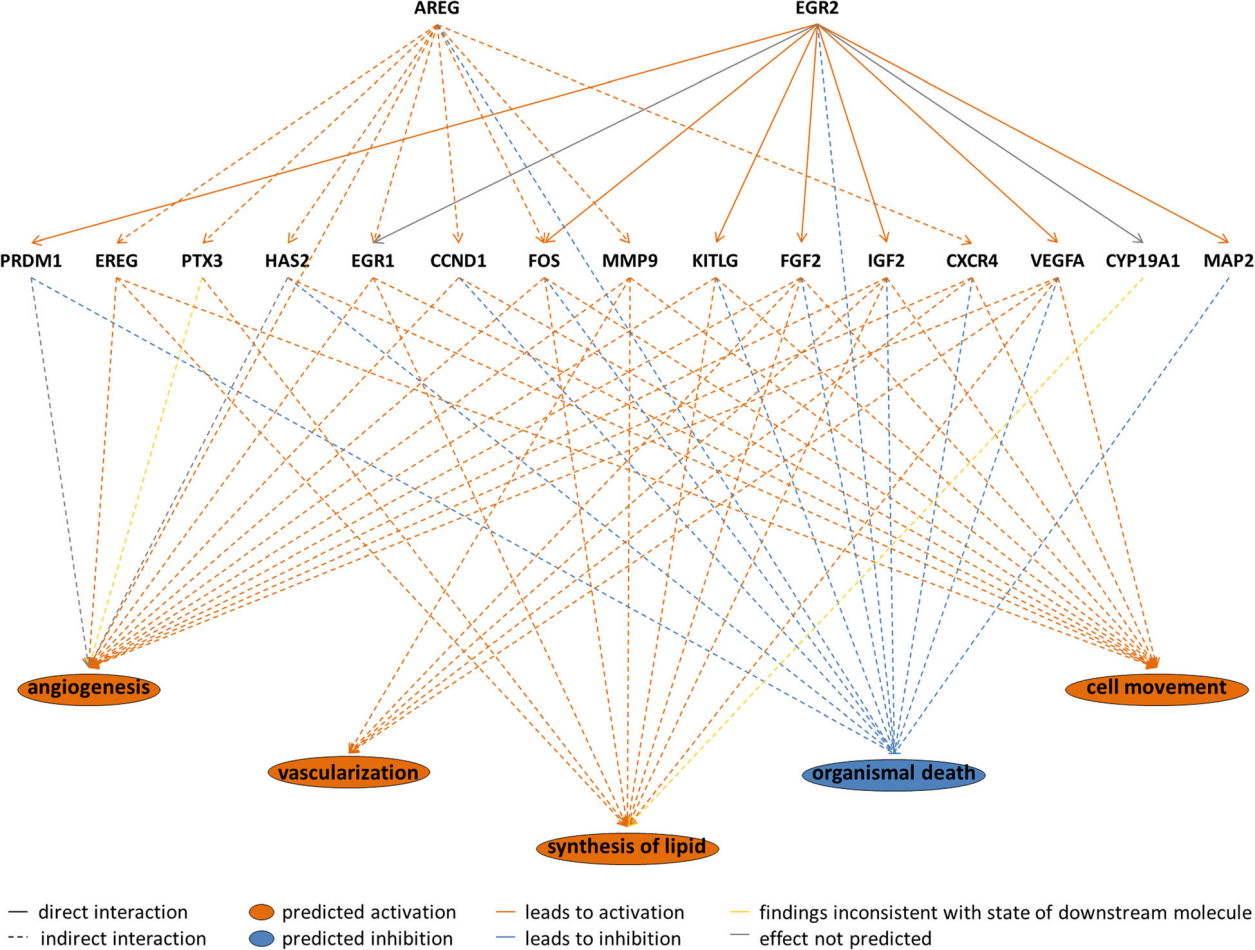
A Regulator Effector Network Analysis by IPA. The analysis combined differentially expressed genes with upstream regulators and biological functions to give hints about potential activation or inactivation (orange or blue)

2193 out of 2429 (i.e. 90.3%) differentially expressed transcript clusters could be assigned to specific genes and mapped to specifically affected pathways and biological functions. Pathway analysis identified the “cAMP-mediated signaling pathway” as well as “Axonal Guidance Signaling” and “TGF-β Signaling” as significantly affected although no prediction regarding activation or inactivation could be made (Fig. 3 and Additional file 1: Table S5). Further analysis identified TNF, beta-estradiol, progesterone and Vegf as major upstream regulators, which might be involved as activating factors and thus responsible for the observed changes of the expression profile (Table 4 and Additional file 1: Table S6). Interestingly, the Regulator Effects analysis of IPA identified a putative activation of the functions “proliferation”, “vascularization”, “angiogenesis” or “cell movement” whereas the biological function “organismal death” was predicted to be inhibited (Fig. 4). This is in accordance with the observation that no significant regulation of pro-apoptotic factors like *CASP4*, *CASP8* or *TP53* (FC -1, 1.18 and − 1.3) could be observed. In this effector analysis AREG and EGR2 were identified as upstream regulators leading to the activation or inactivation of these functions. However, both were not among the top Upstream Regulator candidates (Table 4).

**Table 4:** Upstream regulators identified by IPA comparing lactate with NaCl vehicle control treated GC

Upstream Regulator	Predicted Activation State	Activation z-score	p-value of overlap
TNF	Activated	3.784	2.27E-29
dexamethasone		0	5.65E-26
beta-estradiol	Activated	2.682	4.18E-25
Cg	Activated	3.524	7.12E-24
IL1B	Activated	2.977	1.68E-23
ESR2		1.642	8.62E-23
forskolin	Activated	2.959	3.19E-22
progesterone	Activated	2.292	1.06E-21
tretinoin	Activated	2.311	1.46E-21
Vegf	Activated	4.187	1.71E-21

## Discussion

Our data clearly show that any treatment with either lactate or the NaCl vehicle control significantly changed the global gene expression profiles of cultured GC when compared to untreated controls. However, it is also obvious that L-lactate induced strongest effects: 487 and 788, but only 266 genes were affected by NaCl treatment as compared to untreated cells. The effect of NaCl alone is clearly visible in the PCA of the microarray dataset that separates the vehicle control and untreated cells, revealing the sensibility of the cell culture model to the composition of media. In addition, these data also clearly underline the need to meticulously observe the respective culture conditions in order to ensure reproducibility between experiments also in other cell culture models. However, focusing on markers of GC differentiation specific effects of L-lactate are even more clearly visible without any unspecific changes in the gene expression profile. In any case these data clearly justify our experimental approach to use NaCl treated cells as valid controls to exclude transcripts from the analysis that were affected just due to increased NaCl concentrations. In contrast, cryo-preservation before culture does not alter gene expression. A previous study comparing GC cultured directly or after cryo-preservation revealed no significant differences [22]. However, it is also clear that culturing by itself remarkably changed the gene expression profile of GC (see Fig. 2), thus underlining that cell culture models like that used during the present study can only partly mimic the in vivo situation. This limits the extrapolation of the data obtained in vitro to in vivo conditions.

In earlier studies we could show that various parameters affect GC differentiation in vitro as cell density, hypoxia and the supplementation of L-lactate [16, 23, 24]. In this study we investigated the global change of gene expression by comparing cells treated with L-lactate or NaCl vehicle control. Former established marker genes for differentiation, e.g. *CYP19A1* or *RGS2* showed a specific down- or up-regulation due to L-lactate treatment as described before [16]. Interestingly, the expression of GC identity marker *FOXL2* was not affected by L-lactate thus indicating that L-lactate treatment does not change their identity throughout the culture period as found during treatment with oleic acid [25].

*TXNIP*, coding for the thioredoxin interacting protein, was remarkably up-regulated (FC 21.97) in L-lactate treated GC indicating a role in the L-lactate induced differentiation process. In contrast, when GC were cultured at high cell density we observed the opposite effect and a tremendous down-regulation of *TXNIP* (FC -79.5) [26]. Possibly, the regulation of *TXNIP* might be a sensor for glucose usage and metabolism as it regulates glucose uptake with increased expression reducing glucose uptake [27, 28]. The strongly reduced expression in high density GC culture model might therefore mirror the need of the cells for increased glucose uptake under these “glucose-deficient” conditions, whereas increased expression in the present L-lactate supplementation model could be a consequence of the ample supply with an alternative energy source thus reducing the need for glucose uptake.

Also *H19* gene expression was observed to be significantly up-regulated in L-lactate treated GC (FC 12.4). *H19* is an imprinted gene of which only the maternal allele is transcribed into a long non-coding RNA [29], known to counteract/regulate the transcription of the paternally imprinted gene *IGF2*, an early growth factor that influences the size of the offspring at birth [30]. *H19* expression is mostly abundant in fetal organs, although a moderate expression of *H19* was found in adult ovarian tissue [31]. In an earlier study it was shown that steroid hormones can induce the expression of *H19*, which is therefore highly expressed in hormone sensitive organs [32]. Additionally it was proposed that *H19* expression is high when the organ or tissue is under extensive re-modulation on physiological and morphological levels. Hence, the massive up-regulation of *H19* in lactate treated GC might reflect an initiation of tissue re-organization as it can be found during the folliculo-luteal transition phase.

As a top down-regulated gene in L-lactate treated cells *VNN1*, a GPI-anchored protein with pantetheinase activity, was identified (FC − 2.8). As a regulator of the tissue response to oxidative stress *VNN1* modulates the glutathione store [33]. In *VNN1* knockout mice reduced inflammation and apoptosis could be observed [33]. Within the follicle an increase in *VNN1* expression levels was proposed as an indicator for follicle growth but might also reflect atretic follicles [34, 35]. The down-regulation of *VNN1* in our cell culture model therefore suggests that GC under conditions of increased L-lactate do not have any disposition to atresia. This is also in line with the Regulator Effector Network Analysis predicting inhibition of “organismal death” (Fig.4).

*MYC* was shown to be down-regulated in the L-lactate treated GC compared to the vehicle control (FC − 2.4). Myc acts as a ubiquitous transcription factor, which targets several genes thus enhancing their expression [36]. It was also stated that *MYC* expression declines during differentiation, when a finely tuned reprogramming takes place. Otherwise, the enhancement by *MYC* would lead to uncontrolled proliferation. In this context the down-regulation of *MYC* in GC indicate cellular differentiation processes taking place under increased L-lactate conditions.

Top-affected pathways identified by the pathway analysis were “cAMP-mediated Signaling”, “Axonal Guidance Signaling” as well as “TGF-β Signaling”. cAMP associated pathways contribute to multiple biological processes either under physiological or pathological conditions [37]. Mainly two different intracellular recipients were identified: proteinkinase A (PKA) and the exchange protein directly activated by cAMP (Epac) [37–39]. Earlier it was proposed that both PKA and Epac are involved in the process of luteinization activated by LH [40–43]. In our former study of density-driven differentiation in bovine GC we could also highlight the involvement of the “cAMP-mediated Signaling” pathway [26]. Thus, the results of the present study reflect a LH induced like differentiation of bovine GC upon L-lactate treatment.

The “Axonal Guidance Signaling” pathway likely affected by L-lactate is known to be involved in GC differentiation in connection to cumulus expansion [44, 45]. In particular *NTN1* (netrin-1) was identified during neuron formation. But emerging evidence also exists that netrin-1 is a critical component in vascular regulation [46, 47], as well as in promoting angiogenesis [48, 49], which was also postulated for Netrin-4 in placenta [50]. *NTN1* was down-regulated with a fold change of − 2.1 in L-lactate treated GC compared to the control, which would suggest that vascular regulation or angiogenic processes are not induced. Interestingly, netrin-1 was found to be present in follicular fluid as well as in the theca and granulosa cell layer of swine antral follicles and was proposed to have anti-angiogenic functions [51]. However, whether netrin-1 acts as angiogenic factor or not is still a matter to debate [52, 53]. Our data suggest that *NTN1* is an anti-angiogenic factor regarding the down-regulation and the putative activation of angiogenesis. But the activation of angiogenesis is not only related to down-regulation of netrin-1, moreover, other more prominent factors are involved as well, e.g. *AREG* or *CCND1*. It seems that the final answer of *NTN1* function regarding angiogenesis is not that straightforward in granulosa cells and needs further investigations.

On the other hand the Slit/Robo pathway is associated with the process of axonal guidance. Several subunits of *SLIT* and *ROBO* were present in the microarray dataset but no differential expression of these could be observed. Nonetheless, *SLIT* and *ROBO* expression could be detected in human luteinized GC or in the CL and are regulated by steroid hormones [54]. Previously, we discussed the involvement of NMDA receptors in mediating the L-lactate effects as it was shown for neurons [16, 55]. In the microarray dataset we could identify the expression of several subunits of NMDARs in bovine GC, however without any differential regulation of these. The highest expression could be identified of *GRIN2D* (signal intensity of 4.6–4.8) and *GRIN2C* (signal intensity of 4.0–4.4) similar to another study of bovine GC in vivo and in vitro [56]. NMDARs are important receptors in axonal guidance and synapse formation [57]. However, if L-lactate signaling might be mediated via NMDA receptors in bovine granulosa cells still has to be elucidated.

The upstream regulator analysis implemented in IPA revealed beta-estradiol and progesterone having an activating influence on the L-lactate treated cell culture model. Classically, estradiol elicits a positive feedback on the hypothalamus that regulates the GnRH secretion. GnRH controls the release of the gonadotropins FSH and LH by diverging pulse frequencies. Additionally, a negative feedback mechanism on FSH secretion at the pituitary is also known [58, 59]. Both actions of estradiol trigger the LH surge leading to ovulation. Interestingly, progesterone was also proposed in having an impact on the L-lactate-mediated changes observed in our cell model, although its concentrations did not change during culture. Progesterone, on the other hand, is a critical parameter for the establishment of an active corpus luteum indicating a transition of the GC’s phenotype towards luteinization. However, whereas expression of the key gene of progesterone synthesis *HSD3B1* is very high in fully luteinized GC (i.e. large luteal cells), shortly after the LH surge, during the folliculo-luteal transition phase, its expression is even slightly reduced as compared to that in GC isolated from large dominant follicles [3, 4]. The observation that expression of *HSD3B1* was almost unchanged after L-lactate supplementation (FC 1.04) indicates that the cells are not fully luteinized, but may have yet adapted only to an early post-LH, but pre-ovulatory phenotype. Vegf as upstream regulator as well as TGF-β signaling indicate the activation of angiogenic factors. It is commonly known that angiogenic processes contribute to ovulation and the later formation of the corpus luteum [60, 61]. Also the IPA Effector Analysis revealed “angiogenesis” or “vascularization” as molecular functions to be activated involving the upstream regulator AREG. The function “organismal death” could be identified as inactivated, thus indicating that L-lactate treatment does not affect the viability of the cultured GC. In addition, the transcription of apoptosis markers like *BAX* or *BCL2* was not induced in L-lactate treated cells thus supporting our assumption that the cells are not driven towards atresia. The function “cell movement” could be identified as activated, which is in line with the forthcoming breakup of follicular cell layers and necessary migratory processes during the formation of the corpus luteum.

## Conclusions

Taken together our data provide novel insights into a possible regulatory role of increased concentrations of L-lactate on cells of the granulosa in large follicles during the folliculo-luteal transition. Our data suggest that the biological function of L-lactate in the granulosa cell layer of growing follicle is complex and by far exceeding its role as a product of hypoxic metabolism and energy source. It seems involved in different signaling pathways thus influencing the expression of many different genes. As a commonly known pathway of folliculogenesis our data suggest PKA signaling to be associated with the L-lactate effects. However, we also gathered first hints that NMDAR signaling usually found in neuron physiology might be involved in differentiation processes induced by L-lactate.

## Additional file

Additional file 1: **Table S1.** List of gene-specific primers used in qPCR. **Table S2.** Principal component analysis of microarray dataset. **Table S3.** Principal component analysis of qPCR. **Table S4.** Differential expressed clusters of lactate vs. vehicle control -treated GC. **Table S5.** Affected pathways identified by IPA. **Table S6.** Upstream regulators identified by IPA (*p* < 0.001). (XLSX 131 kb)

## Abbreviations

CL: Corpus luteum
E2: Estradiol
EGF: Epidermal growth factor
FC: Fold change
FDR: False discovery rate
FSH: Follicle stimulating hormone
GC: Granulosa cells
IGF: Insulin-like growth factor
IPA: Ingenuity Pathway Analysis
LH: Luteinizing hormone
P4: Progesterone
PBS: Phosphate buffered saline
PCA: Principal component analysis
qPCR: Quantitative real-time PCR
TGF: Transforming growth factor

## References

[1] Murphy BD (2000). Models of luteinization. Biol Reprod.

[2] Lussier JG, Diouf MN, Levesque V, Sirois J, Ndiaye K (2017). Gene expression profiling of upregulated mRNAs in granulosa cells of bovine ovulatory follicles following stimulation with hCG. Reprod Biol Endocrinol.

[3] Christenson LK, Gunewardena S, Hong X, Spitschak M, Baufeld A, Vanselow J (2013). Research resource: preovulatory LH surge effects on follicular theca and granulosa transcriptomes. Mol Endocrinol.

[4] Nimz M, Spitschak M, Schneider F, Fürbass R, Vanselow J (2009). Down-regulation of genes encoding steroidogenic enzymes and hormone receptors in late preovulatory follicles of the cow coincides with an accumulation of intrafollicular steroids. Domest Anim Endocrinol.

[5] Dias FCF, Khan MIR, Sirard MA, Adams GP, Singh J (2018). Transcriptome analysis of granulosa cells after conventional vs long FSH-induced superstimulation in cattle. BMC Genomics.

[6] Garlanda C, Bottazzi B, Bastone A, Mantovani A (2005). Pentraxins at the crossroads between innate immunity, inflammation, matrix deposition, and female fertility. Annu Rev Immunol.

[7] Salustri A, Garlanda C, Hirsch E, De AM, Maccagno A, Bottazzi B, Doni A, Bastone A, Mantovani G, Beck PP (2004). PTX3 plays a key role in the organization of the cumulus oophorus extracellular matrix and in in vivo fertilization. Development.

[8] Sayasith K, Sirois J, Lussier JG (2013). Expression, regulation, and promoter activation of Vanin-2 (VNN2) in bovine follicles prior to ovulation. Biol Reprod.

[9] Kehrl JH, Sinnarajah S (2002). RGS2: a multifunctional regulator of G-protein signaling. Int J Biochem Cell Biol.

[10] Wu YL, Chuang HH, Kou YR, Lee TS, Lu SH, Huang YC, Nishi Y, Yanase T (2008). Regulation of LH receptor and PGF2alpha receptor signaling by the regulator of G protein signaling 2 (RGS2) in human and mouse granulosa cells. Chin J Physiol.

[11] Sayasith K, Sirois J, Lussier JG (2014). Expression and regulation of regulator of G-protein signaling protein-2 (RGS2) in equine and bovine follicles prior to ovulation: molecular characterization of RGS2 transactivation in bovine granulosa cells. Biol Reprod.

[12] Orisaka M, Tajima K, Tsang BK, Kotsuji F (2009). Oocyte-granulosa-theca cell interactions during preantral follicular development. J Ovarian Res.

[13] Drummond AE (2006). The role of steroids in follicular growth. Reprod Biol Endocrinol.

[14] Jayawardana BC, Shimizu T, Nishimoto H, Kaneko E, Tetsuka M, Miyamoto A (2006). Hormonal regulation of expression of growth differentiation factor-9 receptor type I and II genes in the bovine ovarian follicle. Reproduction.

[15] Knight PG, Glister C (2006). TGF-{beta} superfamily members and ovarian follicle development. Reproduction.

[16] Baufeld A, Vanselow J (2018). Lactate promotes specific differentiation in bovine granulosa cells depending on lactate uptake thus mimicking an early post-LH stage. Reprod Biol Endocrinol.

[17] Leroy JLMR, Vanholder T, Delanghe JR, Opsomer G, Van Soom A, Bols PEJ, de Kruif A (2004). Metabolite and ionic composition of follicular fluid from different-sized follicles and their relationship to serum concentrations in dairy cows. Anim Reprod Sci.

[18] Leese HJ, Lenton EA (1990). Glucose and lactate in human follicular fluid: concentrations and interrelationships. Hum Reprod.

[19] Zeilmaker GH, Verhamme CM (1977). Lactate concentrations in pre-ovulatory follicles of pro-oestrous rats before and after onset of oocyte maturation. Acta Endocrinol.

[20] Perkins JR, Dawes JM, McMahon SB, Bennett DL, Orengo C, Kohl M (2012). ReadqPCR and NormqPCR: R packages for the reading, quality checking and normalisation of RT-qPCR quantification cycle (Cq) data. BMC Genomics.

[21] Ritchie ME, Phipson B, Wu D, Hu Y, Law CW, Shi W, Smyth GK (2015). limma powers differential expression analyses for RNA-sequencing and microarray studies. Nucleic Acids Res.

[22] Baufeld A, Vanselow J. A tissue culture model of estrogen-producing primary bovine granulosa cells. J Vis Exp. 2018;139. 10.3791/58208. 10.3791/58208 PMC6235104 30247464

[23] Baddela VS, Sharma A, Viergutz T, Koczan D, Vanselow J (2018). Low oxygen levels induce early Luteinization associated changes in bovine granulosa cells. Front Physiol.

[24] Baufeld A, Vanselow J (2013). Increasing cell plating density mimics an early post-LH stage in cultured bovine granulosa cells. Cell Tissue Res.

[25] Yenuganti VR, Vanselow J (2017). Cultured bovine granulosa cells rapidly lose important features of their identity and functionality but partially recover under long-term culture conditions. Cell Tissue Res.

[26] Baufeld A, Koczan D, Vanselow J (2017). Induction of altered gene expression profiles in cultured bovine granulosa cells at high cell density. Reprod Biol Endocrinol.

[27] Parikh H, Carlsson E, Chutkow WA, Johansson LE, Storgaard H, Poulsen P, Saxena R, Ladd C, Schulze PC, Mazzini MJ (2007). TXNIP regulates peripheral glucose metabolism in humans. PLoS Med.

[28] Chutkow WA, Patwari P, Yoshioka J, Lee RT (2008). Thioredoxin-interacting protein (Txnip) is a critical regulator of hepatic glucose production. J Biol Chem.

[29] Zhang S, Kubota C, Yang L, Zhang Y, Page R, O'Neill M, Yang X, Tian XC (2004). Genomic imprinting of H19 in naturally reproduced and cloned cattle. Biol Reprod.

[30] Petry CJ, Ong KK, Barratt BJ, Wingate D, Cordell HJ, Ring SM, Pembrey ME, Reik W, Todd JA, Dunger DB (2005). Common polymorphism in H19 associated with birthweight and cord blood IGF-II levels in humans. BMC Genet.

[31] Khatib H, Schutzkus V (2006). The expression profile of the H19 gene in cattle. Mamm Genome.

[32] Adriaenssens E, Lottin S, Dugimont T, Fauquette W, Coll J, Dupouy JP, Boilly B, Curgy JJ (1999). Steroid hormones modulate H19 gene expression in both mammary gland and uterus. Oncogene.

[33] Berruyer C, Martin FM, Castellano R, Macone A, Malergue F, Garrido-Urbani S, Millet V, Imbert J, Dupre S, Pitari G (2004). Vanin-1−/− mice exhibit a glutathione-mediated tissue resistance to oxidative stress. Mol Cell Biol.

[34] Nivet AL, Vigneault C, Blondin P, Sirard MA (2013). Changes in granulosa cells' gene expression associated with increased oocyte competence in bovine. Reproduction.

[35] Girard A, Dufort I, Douville G, Sirard MA (2015). Global gene expression in granulosa cells of growing, plateau and atretic dominant follicles in cattle. Reprod Biol Endocrinol.

[36] Nie Z, Hu G, Wei G, Cui K, Yamane A, Resch W, Wang R, Green DR, Tessarollo L, Casellas R (2012). C-Myc is a universal amplifier of expressed genes in lymphocytes and embryonic stem cells. Cell.

[37] Cheng X, Ji Z, Tsalkova T, Mei F (2008). Epac and PKA: a tale of two intracellular cAMP receptors. Acta Biochim Biophys Sin Shanghai.

[38] Taylor SS, Yang J, Wu J, Haste NM, Radzio-Andzelm E, Anand G (2004). PKA: a portrait of protein kinase dynamics. Biochim Biophys Acta.

[39] Meinkoth JL, Alberts AS, Went W, Fantozzi D, Taylor SS, Hagiwara M, Montminy M, Feramisco JR (1993). Signal transduction through the cAMP-dependent protein kinase. Mol Cell Biochem.

[40] Morris JK, Richards JS (1995). Luteinizing hormone induces prostaglandin endoperoxide synthase-2 and luteinization in vitro by A-kinase and C-kinase pathways. Endocrinology.

[41] Chin EC, Abayasekara DR (2004). Progesterone secretion by luteinizing human granulosa cells: a possible cAMP-dependent but PKA-independent mechanism involved in its regulation. J Endocrinol.

[42] Henriquez S, Kohen P, Munoz A, Godoy A, Orge F, Strauss JF, Devoto L (2017). In-vitro study of gonadotrophin signaling pathways in human granulosa cells in relation to progesterone receptor expression. Reprod BioMed Online.

[43] Leung PC, Steele GL (1992). Intracellular signaling in the gonads. Endocr Rev.

[44] Hatzirodos N, Irving-Rodgers HF, Hummitzsch K, Harland ML, Morris SE, Rodgers RJ (2014). Transcriptome profiling of granulosa cells of bovine ovarian follicles during growth from small to large antral sizes. BMC Genomics.

[45] Salilew-Wondim D, Ahmad I, Gebremedhn S, Sahadevan S, Hossain MD, Rings F, Hoelker M, Tholen E, Neuhoff C, Looft C (2014). The expression pattern of microRNAs in granulosa cells of subordinate and dominant follicles during the early luteal phase of the bovine estrous cycle. PLoS One.

[46] Toque HA, Fernandez-Flores A, Mohamed R, Caldwell RB, Ramesh G, Caldwell RW (2017). Netrin-1 is a novel regulator of vascular endothelial function in diabetes. PLoS One.

[47] Prieto CP, Ortiz MC, Villanueva A, Villarroel C, Edwards SS, Elliott M, Lattus J, Aedo S, Meza D, Lois P (2017). Netrin-1 acts as a non-canonical angiogenic factor produced by human Wharton's jelly mesenchymal stem cells (WJ-MSC). Stem Cell Res Ther.

[48] Xing Y, Lai J, Liu X, Zhang N, Ming J, Liu H, Zhang X (2017). Netrin-1 restores cell injury and impaired angiogenesis in vascular endothelial cells upon high glucose by PI3K/AKT-eNOS. J Mol Endocrinol.

[49] Yang X, Li S, Zhong J, Zhang W, Hua X, Li B, Sun H (2017). CD151 mediates netrin-1-induced angiogenesis through the Src-FAK-Paxillin pathway. J Cell Mol Med.

[50] Dakouane-Giudicelli M, Brouillet S, Traboulsi W, Torre A, Vallat G, Si Nacer S, Vallee M, Feige JJ, Alfaidy N, de Mazancourt P (2015). Inhibition of human placental endothelial cell proliferation and angiogenesis by netrin-4. Placenta.

[51] Baioni L, Basini G, Bussolati S, Cortimiglia C, Grasselli F (2010). Netrin-1: just an axon-guidance factor?. Vet Res Commun.

[52] Castets M, Mehlen P (2010). Netrin-1 role in angiogenesis: to be or not to be a pro-angiogenic factor?. Cell Cycle.

[53] Yang Y, Zou L, Wang Y, Xu KS, Zhang JX, Zhang JH (2007). Axon guidance cue Netrin-1 has dual function in angiogenesis. Cancer Biol Ther.

[54] Dickinson RE, Duncan WC (2010). The SLIT-ROBO pathway: a regulator of cell function with implications for the reproductive system. Reproduction.

[55] Yang J, Ruchti E, Petit JM, Jourdain P, Grenningloh G, Allaman I, Magistretti PJ (2014). Lactate promotes plasticity gene expression by potentiating NMDA signaling in neurons. Proc Natl Acad Sci U S A.

[56] Khan DR, Fournier E, Dufort I, Richard FJ, Singh J, Sirard MA (2016). Meta-analysis of gene expression profiles in granulosa cells during folliculogenesis. Reproduction.

[57] Wang PY, Petralia RS, Wang YX, Wenthold RJ, Brenowitz SD (2011). Functional NMDA receptors at axonal growth cones of young hippocampal neurons. J Neurosci.

[58] Charlesworth MC, Schwartz NB (1986). Estrogen inhibition of LH and FSH secretion: effects of a GnRH antagonist. Am J Phys.

[59] Shaw ND, Histed SN, Srouji SS, Yang J, Lee H, Hall JE (2010). Estrogen negative feedback on gonadotropin secretion: evidence for a direct pituitary effect in women. J Clin Endocrinol Metab.

[60] Schams D, Berisha B (2004). Regulation of corpus luteum function in cattle--an overview. Reprod Domest Anim.

[61] Reynolds LP, Grazul-Bilska AT, Redmer DA (2000). Angiogenesis in the corpus luteum. Endocrine.

